# Social Cohesion and Community Resilience During COVID-19 and Pandemics: A Rapid Scoping Review to Inform the United Nations Research Roadmap for COVID-19 Recovery

**DOI:** 10.1177/0020731421997092

**Published:** 2021-07

**Authors:** Rae L. Jewett, Sarah M. Mah, Nicholas Howell, Mandi M. Larsen

**Affiliations:** 1 7938 University of Toronto, Toronto, Ontario, Canada; 2 5620 McGill University, Montreal, Quebec, Canada; 3 Bremen International Graduate School of Social Sciences (BIGSSS), 84498 Jacobs University Bremen, Germany

**Keywords:** social cohesion, COVID-19, United Nations, pandemics, community resilience, stakeholder engagement, disaster response, social capital

## Abstract

Shock events uncover deficits in social cohesion and exacerbate existing social inequalities at the household, community, local, regional, and national levels. National and regional government recovery planning requires careful stakeholder engagement that centers on marginalized people, particularly women and marginalized community leaders. The aim of this rapid scoping review was to inform the United Nations Research Roadmap for the COVID-19 Recovery, based on Pillar 5 of the United Nations Framework for the Immediate Socioeconomic Response to COVID-19: Social Cohesion and Community Resilience. We present a summary of key concepts across the literature that helped situate this review. The results include a description of the state of the science and a review of themes identified as being crucial to sustainable and equitable recovery planning by the United Nations. The role of social cohesion during a disaster, particularly its importance for upstream planning and relationship building before a disaster occurs, is not well understood and is a promising area of future research. Understanding the applicability of social cohesion measurement methodologies and outcomes across different communities and geographies, as well as the development of new and relevant instruments and techniques, is urgently needed in the context of the global COVID-19 pandemic.

## Introduction

### Social Cohesion and Disaster Recovery

Shock events, whether natural disasters, financial crises, pandemics, or armed conflicts, have lasting social, political, and economic effects on societies and the health of societies. Social cohesion is a critical resource for disaster recovery planning and an important component of the predisaster, acute, postdisaster, and recovery phases.^1^ Different types of shock events co-occur around the world, and the consequences and subsequent opportunities for recovery tend to be inequitably distributed across populations. Both social cohesion and community resilience provide opportunities before a disaster occurs, during the acute phase, and in recovery planning to identify and address inequalities, and they are important for inclusive recovery strategies that support the needs of all in the community.

Social cohesion refers to the degree of social connectedness and solidarity between different community groups within a society, as well as the level of trust and connectedness between individuals and across community groups.^1–3^ It exists on multiple levels of organization, from the household level through international relations, all of which are intertwined and interact with one another.^4^ The relationships among individuals and communities and their local, regional, and national levels of government are affected by social cohesion.

Government funding and programs are often forced to respond to priorities that are shared across entire nations or large geographies at the expense of addressing the needs of vulnerable and marginalized groups. The gap between the needs of local municipalities and the responses and actions of government during a disaster can often drive public mistrust, especially in communities without strong social cohesion and community resilience.^2^ Community engagement and strong social networks are instrumental for identifying priorities and solutions that are more likely to be appropriate, lasting, and supported by the affected community.^1^ Moreover, enabling civic participation allows communities to generate the financial and human capital to identify and prioritize their own goals, which can sometimes be beyond the scope of government intervention.^1–3,5^ Also important is the geographic scale of the priorities being identified, solved, and coordinated. Contested borders and resource allocation are a source of tension across community, local, regional, and national governments. This is further complicated by disputes across levels of community and governmental organizations that can potentially disrupt communities and social cohesion. Often at issue are questions of (1) who holds responsibility for identifying the problems caused by shock events, (2) where leadership should be sought toward devising solutions, (3) who ought to finance recovery efforts, and (4) how interventions will be evaluated. Also crucial is consultations with community stakeholders to make sure the diverse needs of potentially vulnerable groups—who are most adversely impacted by shock events and a lack of social cohesion—are met. Recovery planning is a complex process that is closely tied with social cohesion and community resilience and requires broad-based community participation.

### Social Cohesion and COVID-19

Consistent with other shock events,^5^ COVID-19 has tested the strength of social cohesion and community resilience across geographic levels (household, community, local, regional, and national). COVID-19 has also affected social cohesion across geographic levels, and the strength of social cohesion prior to COVID-19 is likely a strong predictor of recovery. Marginalized communities are also less likely to have access to opportunities and resources for social and economic recovery from pandemics, even though the social capital within these communities may be high.^5–10^ *Social capital*, the degree of interpersonal relationships and connectedness that we rely on for nongovernment aid during a crisis, is dramatically hampered by the very nature of distancing measures required to slow the spread of COVID-19, including events of social and/or cultural significance that build all types of local, national, and international social cohesion, such as weddings, funerals, sporting events,^11^ and knowledge-sharing conferences.^9^ Digital forms of communication have become some of the only means of maintaining social capital, but it should be noted that this technology is afforded to those with the wealth to sustain it, and urban geography allows telecommunication infrastructure that rural areas lack.^12^ Some consider COVID-19 to be the first global pandemic in the information-sharing age, with lessons to learn from the Ebola and Zika virus epidemics of 2016.^5^ These lessons primarily include creating formal opportunities for stakeholder engagement and community resilience before a disaster so that strong social cohesion between public and government is in place to enable an appropriate and quick response, as well as to maintain communication during times of crisis.^13,14^

In this paper, our aim was to perform a scoping review of the literature on relationships between social cohesion and disaster recovery, with special attention paid to pandemics. This was performed to support the United Nations Research Roadmap for the COVID-19 Recovery. The results of this review will help identify ways in which social cohesion influences disaster recovery, and gaps in current research.

## Summary of Key Concepts

### Social Capital

Social capital is the most studied concept related to social cohesion within the disaster recovery literature. This concept was coined by Robert Putnam, who defined it as “the features of social organizations, such as networks, norms, and trust, that facilitate action and cooperation for mutual benefit.”^4^ Social capital has been identified as a key driver of sustainable disaster recovery. Although social capital relies heavily on preexisting positive connections and relationships across households, communities, and levels of government, there are many examples of social capital growing out of local community response to a crisis.^15^ During a disaster and in the disaster recovery phase, social capital acts as a resource that often supplements the work of local, regional, and national governments. Social capital is an important consideration for long-lasting disaster recovery, and it affects the ability of local community groups to identify community vulnerabilities, marginalized groups, priorities, and appropriate solutions that could be more relevant and feasible than those proposed by national or regional government bodies.^9,15–19^

Social capital has been conceptualized as having three types.^20^ *Bonding social capital* comprises the social ties linking people who share sociodemographic characteristics. Bonding social capital tends to reinforce conformity and solidarity and can be exclusive to others who do not share similar characteristics or do not conform to the norms of the group. *Bridging social capital* is the social ties that links people across and between social differences and divides. This type of social capital tends to be more inclusive and enables people to link to different types and sources of resources, crowdsource, and share important information. Last, *linking social capital* is the social ties between people based on their access to power and resources, including the wealthy or those with political influence.

More heterogeneous communities have greater bridging social capital, which enables exposure to and the development of new ideas and innovation. Bridging social capital tends to enhance social cohesion, whereas more homogeneous communities (which tend to have stronger bonding social capital) are believed to have lower because social cohesion.^15^ The modern age's telecommunications, travel, and overall globalization have advanced the bridging social capital of many communities and nations. However, more culturally and demographically homogeneous populations may not have the same response to these developments and may continue to rely on bonding social capital.^21^ Additionally, disaster events often alter the demographic makeup of communities, regions, and even nations, a phenomenon that has been observed as a result of evacuations during tsunami, earthquakes, and hurricane events.^22^ The nature and extent to which pandemics alter the geodemographic makeup of populations and change migration patterns are known to disproportionately affect the elderly, children, those in poverty, and racialized populations.^8,23^ This phenomenon can drive the advancement or degradation of social cohesion through changes in neighborhoods, communities, and regions, and it can bring about shifts in the makeup of communities, as well as the lifestyle and sociocultural practices of people. Linking-type social capital remains understudied and unmeasured in the disaster literature. However, those with greater power and access to resources are more likely to benefit from social capital of this variety, which may be a consideration for equitable recovery planning and the need for national and regional stakeholder engagement planning that centers on local communities.

Mutual aid among communities often depends on the willingness of those with financial, institutional, and political power to share those resources during a crisis. Faith-based groups are recognized as a source of bonding social capital for individuals lacking household social capital, and they have played important roles in local disaster response (eg, Hurricane Katrina) by providing basic services, psychosocial support, and conflict resolution.^24,25^ However, stakeholder engagement and aid allocation through systems controlled by faith-based groups can be problematic, because they are member-limited organizations that may impose certain values and beliefs that do not align with the communities they aim to serve.^24–26^ Some concerns include the possibility of coercion, provisions that are conditional on compliance with worldviews, and the potential exclusion of local marginalized groups.^25^

Grassroots initiatives often emerge during a disaster and in the recovery phase of a disaster, particularly in the absence of communication and aid from the government. Hurricane Katrina (2005) was an extraordinary example of local social capital, where the public acted to evacuate and respond^24,27,28^ in the absence of stakeholder engagement and action from the state and the federal government. In other scenarios, such as the Christchurch earthquakes in 2010 and 2011, community members reported feeling left out of government interventions, even though the New Zealand government acted quickly.^18,29^ The widespread feelings of exclusion have been linked to the telecommunication infrastructure that had been disabled during the event due to the natural disaster, with no plan for face-to-face consultation being made with affected residents.

### Community Resilience

*Community resilience* during a disaster refers to a community's degree of adaptability to changing circumstances and challenges.^30^ During the acute and recovery phases of a disaster, *adaptability* refers to a community's capacity to identify needs as they evolve and apply the necessary resources to address those needs (financial, social, labor force, skills, etc). Within the context of the disaster literature, support for community resilience has grown. This is due in part to the acknowledgment that social capital is a critical resource for recovery. Additionally, due to the focus on communities, rather than individuals. Individuals are limited in their ability to respond in a crisis, restricted to resources that made available and actionized at this scale. Communities can draw from collective individual resources as well as resources and opportunities available only at aggregated scales of cooperative organization.^31^ Community resilience is also a critical resource for governments to consider in their recovery planning. Community resilience can strengthen and support recovery in many ways, such as, including additional finances, public trust, and public support for policies. As community resilience requires social capital, it is often used as a proxy for community and regional-level social capital.^31^

The Australian, Canadian, and US governments have disaster response plans that include a resilience framework.^32–34^ Regional and national governments have increasingly recognized the ability of communities to self-organize during a disaster and have made efforts to avoid the “command and control” approach commonly taken in previous disasters.^35^ The importance of community resilience has also been noted for the Ebola epidemic in Liberia. Strong leadership, tight bonds, and sense of kinship at the community level; trusted communication channels; and trust among stakeholders were key factors identified in qualitative interviews^13^ that facilitated collection actions within communities that directed government recovery responses.

The Index of Perceived Community Resilience is a composite measure that includes indicators such as physical environment, level of neighborhood help, isolation from the rest of the region, the openness of ideas, pride, community leaders, and more.^36^ This index has been used in several studies, has been through multiple iterations, and has been applied to different disaster types, such as wildfires and floods. Future research will likely be aligned with the body of work on social capital and suggests the need for further investigation into the connections between individual and community resilience, critical evaluation of the indicators chosen, and community-led selection of indicators. Similarly, future work should seek to understand the appropriateness of this model for different disaster types (such as pandemics), different geographic scales, regions of different income status, as well as the challenges pertaining to autocratic governance and armed conflict.^36^

### Community Engagement

*Community engagement* refers to activities that build trust between all levels of government and its constituents.^37^ These activities can include different forms of communication, community events, information-sharing technologies, and more. The ways in which communities engage vary widely around the world and depend heavily on the lifestyle and cultural practices of the community; however, one common theme centers around respect for lived experience, individual and community priorities, and diversity of the public in balance with the mandate and interests of those holding government positions.^14,38^ Trust between community members and all levels of government is a critical element of social capital.^15,16,19,27,39,40^ Types of community engagement that are thought to undermine trust during a disaster include (a) top-down paternalistic governance, (b) undermining of social networks, (c) inefficient social institutions, and (d) inciting social divisions.^22^ Stakeholder engagement facilitates the identification of recovery needs, dynamic exchange of information, and consolidation of diverse perspectives. Although at first, it is time consuming, it will save time and provide lasting benefits in the recovery process.^28^ The benefits of community engagement to the recovery process include the opportunity to better tailor policies to match public priorities, understanding of diverse need, attention to nuanced circumstances, public buy-in and participation in policies, and improved appropriateness of interventions that, in turn, increases efficiency and cost effectiveness.^1,18,26,35,38^

Beyond the communication of transmission risk, we have learned about the importance of holistic community engagement from previous epidemics.^29^ A 5-step community engagement process was developed from the Ebola crisis and includes: (1) understanding the community, (2) providing relevant information across communities, (3) feedback engagement, (4) understanding changing needs, and (5) centering communities to lead and codevelop programs.^14^ Exclusively relying on digital telecommunications may exclude those without such devices, low-resource regions, as well as rural areas with limited infrastructure, including informal urban settlements. Moreover, many qualitative studies report a preference for face-to-face opportunities for stakeholder engagement.^18^

The results of community engagement with marginalized groups during COVID-19 are becoming increasing available. The recommendations flowing from these processes have urged communities and government to continue to engage with community leaders and co-identify vulnerable groups in their communities. There are calls to prioritize community engagement with certain groups expected to be particularly affected by the COVID-19 pandemic, including indigenous peoples, informal workers, people without shelter, incarcerated individuals, institutionalized individuals, residents of informal urban settlements, people with mental health conditions, people affected by addiction and drug use disorders, and stateless people.^38,41–46^

## Methods

Keywords and themes were identified from Pillar 5: Social Cohesion and Community Resilience (Table 1). We chose Web of Science as the primary peer-reviewed literature database source for its indexing of targeted social science journals. A search strategy was developed and implemented with final records identified on August 6, 2020 (Table 2). Records were reviewed for inclusion by title/abstract using the criteria in Table 3. We also evaluated gray literature sources using the same keywords. Records were then reviewed by full text, along with the title and abstract.

**Table 1.** List of Keywords and Themes from United Nations Framework for Socioeconomic Response to COVID-19: Pillar 5.

Keywords	Themes
Social cohesion Community resilience Social capital Networks of relationships Social dialogue Democratic engagement Political engagement Peaceful assembly Freedom of association Collective bargaining Freedom of expression Informal work sector Conflict Natural disaster Women leaders Political transitions Public freedom Threats on privacy Free speech Overreach of emergency powers Compliance with national human rights institutions	Community-led, participatory planning, local oversight Digital engagement platforms and access to information/organization Geospatial data collection techniques for informal urban settlements vulnerable to COVID-19, disease, and shocks to system to predict movements from urban poor in distress seeking refuge in their rural areas of origin^27^ City resilience and urban profiles, generate an understanding of how the response can be tailored to complex urban systems, relate to the built environment, and connect community level and urban resilience^28^ Innovative community engagement practices such as mass media, digital media, and local arts and culture Information dissemination such as open-source software used to create chatbots, interactive voice response virtual support mechanisms, hotlines, and instant messaging services, particularly for women The resilience of cities (and converse risk) and communities to withstand shocks from an economic downturn or natural disasters Global mapping of encroachment, illegal trade, and wet markets that are pathways for pathogen transmission Ecosystem encroachments and harmful practices, ecosystems, and illegal trade, while protecting communities that depend on them for food supply and livelihood. Connected to existing CITES and CMS guidance and CDS COP15

**Table 2.** Search Strategy, Web of Science (August 6, 2020) Restrictions: 1990 to 2020, English Language, Social Sciences Databases, Articles Only.

Line	Topic	Terms	No. of records returned
1	COVID-19	Coronavirus OR betacoronavirus OR coronavirus infections OR dnCOV OR 2019nCoV OR 19nCoV OR COVID19 OR COVID OR SARS-COV-2 OR SARSCOV-2 OR SARSCOV2	2,059
2	All pandemics	Epidemic* OR pandemic* OR SARS OR “severe acute respiratory” OR SARS-CoV-1 OR SARS-CoV OR SARSr-CoV OR SARSCoV OR “Middle East respiratory syndrome” OR MERS OR “camel flu” OR MERS-CoV OR MERSCoV OR ebola* OR “influenza A” OR H1N1 OR “swine flu”	20,996
3	Shock events and disaster recovery	“Disaster recovery” OR “shock event” OR “post-disaster” OR “natural disaster” OR earthquake OR tsunami OR flood OR hurricane	20,936
4	COVID-19 or pandemics	1 or 2	21,471
5	COVID-19, pandemics and shock events	1 OR 2 OR 3	42,175
6	Pillar 5 and SDG #16 keywords	“Sustainable development goal*” OR “SDG” OR “armed conflict” OR homicide OR propaganda OR “freedom of information” OR persecut* OR prison OR arrest* OR incarcerat* OR “social cohesion” OR “community resilience” OR “social network*” OR “social identity” OR “social capital” OR “social dialogue” OR “democratic engagement” OR “political engagement” OR “peaceful assembly” OR “freedom of assembly” OR “collective bargaining” OR War OR “Civil war” OR protest* OR “women leaders” OR “free speech” OR “freedom of speech” OR “emergency powers” OR “human rights” OR “national human rights institution*” OR “community led” OR “local engagement” OR “local oversight” OR “participatory planning” OR “digital engagement” OR “digital platforms” OR refugee OR “city resilience” OR “urban resilience” OR “rural resilience” OR “built environment” OR “community engagement” OR “mass media” OR “local arts” OR “information dissemination” OR “virtual support” OR hotline OR chatbot* OR “instant messag*” OR “citizen Engagement” OR “stakeholder engagement” OR “engagement process” OR exodus OR “informal urban settlement*” OR “predict movement*” OR “map* of encroachment” OR “illegal trade” OR “wet market*” OR “ecosystem encroachment” OR “culture of science” OR “conspiracy theor*” OR “social license”	217,990
7	Pillar 5 keywords and COVID-19	1 AND 5	161
8	Pillar 5 keywords and pandemics	2 AND 5	2,270
9	Pillar 5 keywords and disasters	3 AND 5	2,583
10	Pillar 5 and COVID-19/pandemics	(1 OR 2) AND 5	2,306
11	Pillar 5 keywords and all shock events (COVID-19, pandemics, shock events)	(1 OR 2 OR 3) AND 5	4,848

**Table 3.** Inclusion/Exclusion Criteria.

Inclusion criteria	Exclusion criteria
Papers regarding social cohesion and community resilience during shock events (pandemics, disasters, etc) from any country Written in English Published Between 1990 and 2020 (1990 was chosen as a reasonable parameter date that captures the modern age of information sharing, modern disasters, shocks, and pandemics) Methodologies include observational commentaries, frameworks, conceptual models, literature reviews, and empirical evidence Papers discussing interactions between humans (as opposed to psychology and mental health epidemiology) Recovery focus	Nonhuman subjects Articles on “disaster recovery” in information technology and computing General shock event/disaster recovery plans for physical infrastructure rebuilding of damaged property Focused on mental health outcomes such as addictions and depression Acute interventions unless they have observed and reported links to social cohesion and community resilience Community resilience in a physical/structural sense to future physical damage (eg, preparing the community for future resilience to flooding would entail physical infrastructure intervention) Exclude individual opinion commentary about political leaders Domestic abuse, child abuse, and intimate partner violence Adherence and compliance to government COVID-19 spread intervention policies Literature on vaccines and antivaccine behaviors and perceptions Tourism after a disaster—pandemic Risk of infectious disease communication strategies Attacks on health care workers and foreign health care worker related literature Artificial intelligence chatbots specific to delivering virtual care

Papers were excluded at the full-text review phase for the following reasons: health system focus without reference to Pillar 5, the resiliency of physical infrastructure, focus on population compliance with pandemic nonpharmaceutical interventions, infectious disease control, project management, commentary with no evidence, political opinion, individual mental health epidemiology, and vaccinations.

## Results

### Social Cohesion: State of the Science

Within the literature on disaster recovery, social cohesion was identified as a primary resource for communities to draw upon during a crisis. The importance of social cohesion is largely driven by the role that social capital plays in fostering social cohesion, with particular attention to elements of social capital and cohesion such as collaborations and stakeholder engagement between community groups and with levels of government, as well as among levels of government. Studies predominantly focused on the rebuilding of infrastructure as an outcome of interest. Many empirical studies aimed to characterize and quantify social cohesion through interviews and surveys, and used broader aggregate methods of data collection. The current state of science on social cohesion research within disaster recovery literature includes the addition of socioeconomic factors as a secondary outcome. This is to be expected, considering the majority of disasters and shock events in recent history have been natural disasters and armed conflicts that cause widespread property damage and mass migration. The most commonly identified method for assessing social cohesion was qualitative interviews.^13,18,22,24,28,29,31^ Some studies employed Buckner's index of cohesion (psychological sense of community, neighborhood attraction, and neighboring).^2^ This index was developed based on a study of cohesion following flooding in various rural Canadian towns and includes self-reported factors such as friendship, belonging, access to help in an emergency, advice, borrowing, and remaining a resident.

Studies used a variety of other methods and approaches for measuring social cohesion and its impacts. One commonly used approach is to model the relationship between variables related to elements of social capital during a disaster (eg, physical health, emotional distress, homeownership, and civic engagement) and different outcomes that represent social capital such as participation in recovery activities (namely, the 2005 US Hurricane Katrina and the 2020 Haiti earthquake).^27^ Another method to measure social cohesion impacts on disaster recovery is surveying self-reported cohesion/recovery with characteristics such as civic engagement, contact with neighbors, and trust, which were reported as factors for faster recovery following the 2012 Indiana (US) tornados.^39^ A different survey regarding a 2011 flood in Australia found that individual bonding social capital moderated postdisaster consequences and cited a need for further research on community-level social capital.^17^ Finally, some studies use social media geotagging and found successful information sharing and recovery planning during the US Hurricane Harvey in 2017; however, the wealth inequity of relying on this source of data should be considered.^3^ Some evidence exists that reviews national disaster recovery plans (which are largely based on infrastructure rebuilding) to understand opportunities to update these plans to include stakeholder engagement processes.^34,47^ However, the lack of large-scale empirical evidence on social cohesion, concomitantly supported by qualitative studies to account for the diverse conditions and life circumstances faced by distinct communities, limits the ability of this research to comprehensively inform and advance global and national recovery planning. Many emphasize the importance of stakeholder engagement in this area of work and research.^1,14,18,19,28,29,36–40,42^

This body of literature has many common themes that agree on factors that promote social cohesion in disaster recovery. They include (a) government interventions (financial stimulus, social protections, etc), (b) government protocols for communicating with the public and stakeholder engagement, (c) community-led and localized recovery efforts, and (d) community-building events and opportunities. Similarly, this body of literature states common themes in factors that impede social cohesion during disaster recovery: (a) armed conflict, (b) government centralization of power, (c) overuse of police, (d) inequitable and unevenly distributed resources, (e) narrowly defined recovery programs at the national level, and (f) lack of stakeholder engagement across levels of government.

### Equity and Social Cohesion

Disasters are often not the direct cause of socioeconomic inequities but uncover and worsen existing disparities.^48^ The United Nations names four major areas of equity: gender, race, income status, and environmental sustainability. Although this review attempted to characterize each area, we note that there is a dearth of evidence on the role of racial inequity and environmental sustainability in the disaster response literature. Evidence on countries of varying income status was also lacking. Therefore, the majority of this section focuses on the role of gender equity.

Gender equity is a fundamental component of social cohesion for any society. “Gender” refers to socially constructed roles and systems that are assigned to men and women. Gender roles and barriers are continually evolving, and are influenced by local faith, culture, and social norms. Social norms heavily dictate gender-based opportunities and limitations in access to resources such as employment, health care, and safety. These factors, plus many others, become critical in the acute and recovery phases of a disaster given their direct link to wealth and social capital, predicting how well households can adapt and recover.^49^ The role of gender equity in the recovery response to any disaster has been widely recognized and prioritized by humanitarian organizations.^6,23^ Yet, there is a lack of theoretical analysis of gender in disaster literature, which poses a challenge for gender-responsive disaster recovery policies.^23^ This is further challenged by a lack of data governance and disaggregation by gender; for example, the Emergency Events Database does not disaggregate by gender, which further exemplifies the failure to recognize the importance of gender in disasters.^48,49^

It is well documented that natural disasters kill more women than men.^50^ Beyond the direct impacts of disasters, gender-based violence increases during and after disasters, which is in part a result of preexisting social, economic, and political inequalities between women and men.^51^ Women face inequitable access to material resources and power, relative to men, which undermines their capacity to recover from a disaster. Moreover, women are often placed in the role of providers and caretakers of children and of the elderly, and often shoulder the burden of caring for the sick.

The evidence gender inequalities pertaining to social cohesion during disasters involves qualitative interviews and social media accounts of the 2010 Chile earthquake and tsunami. This study reported 5 stages corresponding to changes in living conditions and power relations, which ultimately demonstrates how the vulnerability of women was intensified after the disaster and how resilience contributed to lessening gender-based vulnerabilities.^48^ The 5 identified stages are: (1) predisaster (traditional gender roles and limited access to resources), (2) gendered disaster evacuation (lack of external aid), (3) emergency (vulnerability, leadership, and grassroots women's organization), (4) recovery (multiple burdens, leadership, and support networks), and (5) postdisaster (long-lasting changes and women's rights movement).^48^ This study demonstrated gender equity and community resilience through women's leadership in grassroots movements as a fundamental and lasting element of gender-based community resilience and social cohesion, which is a common theme identified in this scoping review.^6,23,48–50^ Several of the included qualitative and quantitative studies consider gender inequity by disaggregating gender in data collection and analysis. Subsequent reporting included gendered labor force themes. Most included studies did not incorporate other equity stratifiers such as racism, environmental sustainability factors, or applicability across low-, middle-, and high-income countries.^22^ For example, Buckner's index of cohesion does not consider racial equity or applicability across countries in the development or selection of indicators.^2^ One study collected data on race but did not make interpretations.^24^

There is a link between informal urban settlements (sometimes referred to as “slums”) and gender equity in disaster response, and the impacts of gender in this context are growing in the literature.^41,43^ There is an increased care burden for women and girls and a diversion of resources from gender protection programs. Moreover, there are increased reports of gender-based violence within informal urban settlements during COVID-19 quarantine, which follow patterns from previous outbreaks such as the Ebola virus.^10^ Despite this gender equity concern, it should be noted that many informal urban settlements are highly organized with a range of local groups and community structures that provide advocacy and services as well as collect data on their populations.^52^ These groups are well situated to lead their own COVID-19 recovery planning and should be considered leaders in their own response to coorganize through local engagement.^43^ It should be noted that there is disagreement in the literature on the quality of data governance for informal urban settlements and the need for intervention, which requires further research.^41,42^

Some commentaries on the environmental impacts on wet markets with respect to COVID-19 and pandemics were identified.^53–55^ These commentaries discuss some perspectives and misinformation that wet markets carry the responsibility for the spread of COVID-19. These commentaries emphasize that wet markets are an essential part of the food supply chain for these communities and play an important cultural and social cohesion role as well. Moreover, these commentaries remind us that such claims of blame are unfounded, as the spread of COVID-19 has been taken up around the world by unmeasurable sources and exacerbated through countless and varied mechanisms.

### Sustainable Development Goal #16 and Social Cohesion

The United Nations Sustainable Development Goals (SDGs) include Goal #16: Peace, Justice, and Strong Institutions, which directly correlates with social cohesion and community resilience during a disaster.^56^ This SDG is evaluated based on many different indicators, including the number of people fleeing war, persecution, and conflict; statehood; and birth certificates. COVID-19 puts SDG #16 priorities at risk through the amplification of social cohesion issues within all countries regardless of low-, middle- or high-income status. In March 2020, the United Nations Security Council made an appeal for a global ceasefire to allow for the safe delivery of humanitarian aid and opportunities for democracy; the extent of compliance is unknown.

Evidence from the literature included in this scoping review regarding SDG#16 includes the above-mentioned opportunities to understand and evaluate social cohesion and community resilience, predominantly through stakeholder engagement that centers on marginalized groups and facilitates self-organization of grassroots initiatives, particularly led by people identifying as women. Additional evidence from the literature regarding SDG#16 and social cohesion during a disaster is presented with themes on collective bargaining, armed conflict, the division between the public and government, and the effects on populations that are incarcerated.

One paper on collective bargaining during COVID-19 was identified that documented the negotiations between American graduate assistant education unions and their employer and recommended: (a) initiating early with leadership, (b) mobilizing union members and the workforce, (c) prioritizing a shortlist of issues relevant to the crisis, and (d) formalizing impact bargaining agreements.^57^

Armed conflict alongside other disasters must be a top international, humanitarian priority. The force multiplier of this complex circumstance directly causes the loss of life and the exponential delay of recovery, which may be decades or generations away. It is well understood that regions of armed conflict will worsen the conditions for the spread of infectious diseases. The most notable example in modern history is the human immunodeficiency virus (HIV)/acquired immunodeficiency syndrome (AIDS) epidemic, which is believed to have started in 1982 in South Africa when the country was engulfed in the social cohesion priority of apartheid (system of racial segregation in place since the 1950s) and the government and community focus was on racial inequity, which allowed for the unintentional deprioritization of HIV/AIDS management. The HIV/AIDS epidemic quickly affected the world and remains a serious threat in subSaharan Africa. Barriers to control HIV/AIDS in this region are attributed to ongoing armed conflict and government instability.^58^ In the present day, many countries and regions around the world battle COVID-19 alongside existing armed conflict and political instability. One example is Yemen, which since March 2015 has endured deadly airstrikes and bombardments, killing more than 100,000 people, 25% of whom are women and children.^7^ Several communicable diseases have flourished in Yemen as a result of this armed conflict, including cholera, dengue, and measles. According to the World Health Organization (WHO), only 51% of health care facilities in Yemen are operational, including a total of 3 COVID-19 testing facilities for the entire country.^7^

### Division Between Public and Government

The division between the public and government is of concern during a disaster and is largely in the hands of the government. This relationship is often cited as a key to social cohesion, with central concepts being inclusive leadership, legitimacy of authority, shared identity, and common goals.^59^ It is documented that tensions can arise when limits are imposed on everyday freedoms. No evidence was found that supports the common belief that misinformation, conspiracy theories, or mandatory masks during a pandemic have been the cause of violent riots or significant loss in social capital. Instead, the literature cites heavy-handed policing of overly stringent and out-of-touch pandemic interventions as well as opportunist populist leaders scapegoating minority groups and suppressing democratic governance as the source of mass public protest during COVID-19.^59–61^

Government practices considered damaging to social cohesion during COVID-19 in countries including the United States, Hungary, Italy, Brazil, and France include overreach of government powers, government support of antilockdown protestors, promotion from national leaders of division between themselves and local and state leaders, exclusionary policies, suspension of civil liberties, lockdown requirements difficult for ethnic minorities to adhere to, limiting the power of legislatures and centralizing power to single offices, and reducing accountability in government.^59–61^ This body of literature generally hypothesizes that disasters have historically provided the opportunity for autocratization through the guise of emergency response, capitalizing on the weaknesses in social capital and cohesion of the public.

### Incarceration and COVID-19

Incarcerated population presents a challenge unique to COVID-19 that is unlike other disasters. With natural disasters, it is often possible for populations that are incarcerated to remain or for the entire population to require evacuation. In the case of COVID-19, there is considerable contention around population density, overcrowding, public health practices, and judicial reform from several countries.^62–67^ Populations that are incarcerated have international human rights to public health care and living standards; however, their sentencing and stay are determined by the detaining state. Despite a WHO call for the immediate and rapid reduction of the population that is incarcerated, countries that most suffer from this issue have failed to comply. Italy experienced protests among its population that are incarcerated, which is currently at 121.75% capacity.^62^ Many American states (California, Ohio, Kentucky, Texas) have released people who are most at risk for COVID-19, such as the elderly or those awaiting sentencing, to house arrest. Other countries that have adopted reduction policies include Albania (house arrest), Brazil (based on the threat to the community), Afghanistan (to release 10,000 people), Indonesia (18,000 people), Ethiopia (4,000 people with <1 year remaining), and Zimbabwe (<36 months remaining in sentence).^62^ Brazil currently has 748,000 people incarcerated and faces the same debate as most other countries, which is balancing releasing people who are vulnerable to COVID-19 with those who do not pose a danger to the community.^63^ One issue across many countries is the lack of reporting of the population that is incarcerated as part of the entire population count of COVID-19 statistics, with little consideration for the open system that links these institutions through staff, health care workers, and people who are often marginalized.^63^

## Discussion/Conclusion

This rapid scoping review has compiled evidence for social cohesion and community resilience during a disaster, particularly within the context of a pandemic, highlighting findings for COVID-19. This rapid review summarized keywords from the United Nations Framework for Socioeconomic Recovery (Pillar 5) to search Web of Science from 1990 up to August 6, 2020. This rapid scoping review was completed to inform the United Nations Research Roadmap for COVID-19 Recovery (November 2020). Due to the rapid nature of knowledge synthesis, this review has some methodological limitations to be considered. This includes the use of one database for identifying sources and one reviewer for article screening and data extraction. Additionally, given the broad arrange and types of literature cited, we were unable to apply a quality assessment to the literature reviewed. Although identified in the search strategy, this scoping review did not discuss articles related to misinformation and conspiracy theories, which is an important topic with respect to social cohesion and community resilience during a disaster that future reviews should explore.

This review included early findings from COVID-19, and repeating this review will be useful to include additional information related to social cohesion and COVID-19 that will emerge as the pandemic evolves globally. The key findings from this rapid review demonstrate that social cohesion and community resilience are important resources in the recovery after any disaster. Investment in social cohesion and community resilience during peaceful and prosperous times is critical to strengthening and leveraging these resources during a crisis. Investment can take many forms, and the local culture and practices in different regions need to be considered. During a disaster, inequities in society are revealed and exacerbated. The identification of inequities and priority setting for intervention and aid benefits from strong social cohesion practices. These practices can include stakeholder engagement protocols, communication plans between government and the public, careful evaluation of government measures that affect public freedoms, and local–regional context. All these activities, and more, must center on marginalized groups including but not limited to women, children, indigenous peoples, racialized peoples, and people with varying abilities. Any protocols or interventions that seek to intervene in social cohesion and community resilience must do so with an equity lens that brings together all parties involved.
